# A New Antifungal Agent (4-phenyl-1, 3-thiazol-2-yl) Hydrazine Induces Oxidative Damage in *Candida albicans*

**DOI:** 10.3389/fcimb.2020.578956

**Published:** 2020-10-07

**Authors:** Quan-Zhen Lv, Ting-Jun-Hong Ni, Li-Ping Li, Tian Li, Da-Zhi Zhang, Yuan-Ying Jiang

**Affiliations:** ^1^School of Pharmacy, Second Military Medical University, Shanghai, China; ^2^Shanghai Tenth People's Hospital, Tongji University School of Medicine, Shanghai, China

**Keywords:** *Candida albicans*, antifungal agent, (4-phenyl-1, 3-thiazol-2-yl) hydrazine, ROS, DNA break

## Abstract

A gradual rise in immunocompromised patients over past years has led to the increasing incidence of invasive fungal infections. Development of effective fungicides can not only provide new means for clinical treatment, but also reduce the occurrence of fungal resistance. We identified a new antifungal agent (4-phenyl-1, 3-thiazol-2-yl), hydrazine (numbered as 31C) which showed high-efficiency, broad-spectrum and specific activities. The minimum inhibitory concentration of 31C against pathogenic fungi was between 0.0625-4 μg/ml *in vitro*, while 31C had no obvious cytotoxicity to human umbilical vein endothelial cells with the concentration of 4 μg/ml. In addition, 31C of 0.5 μg/ml could exhibit significant fungicidal activity and inhibit the biofilm formation of *C. albicans*. *In vivo* fungal infection model showed that 31C of 10 mg/kg significantly increased the survival rate of *Galleria mellonella*. Further study revealed that 31C-treatment increased the reactive oxygen species (ROS) in *C. albicans* and elevated the expression of some genes related to anti-oxidative stress response, including *CAP1, CTA1, TRR1*, and *SODs*. Consistently, 31C-induced high levels of intracellular ROS resulted in considerable DNA damage, which played a critical role in antifungal-induced cellular death. The addition of ROS scavengers, such as glutathione (GSH), N-Acetyl-L-cysteine (NAC) or oligomeric proanthocyanidins (OPC), dramatically reduced the antifungal activities of 31C and rescued the 31C-induced filamentation defect. Collectively, these results showed that 31C exhibited strong antifungal activity and induced obvious oxidative damage, which indicated that compounds with a structure similar to 31C may provide new sight for antifungal drug development.

## Introduction

In recent years, the usage of immunosuppressive agents and the increasing numbers of immunodeficiency patients and severe infections has led to growing incidence of fungal infections (Pappas et al., 2018). However, the limitations of fungal diagnosis and antifungal drugs result in the high mortality rate of systemic fungal infection as 20–50% (Kullberg and Arendrup, 2015; Papon et al., 2020). In addition, some fungal clinical isolates have developed resistance to the commonly used azoles and echinocandins (Demers et al., 2018; Revie et al., 2018). Therefore, the development of broad-spectrum drugs with new antifungal mechanisms is an urgent request for treating fungal infections.

Many new antifungal scaffolds validated by different assays were discussed in detail by Na Liu et al. As reviewed, they mainly focused on high-throughput screening, drug repurposing, antifungal natural products and new antifungal targets. At the same time, they referred a guideline for the ideal antifungal agent, which should have fungicidal activity, a broad spectrum and good selectivity toward a fungal-specific target (Liu et al., 2016). More importantly, powerful fungicides can effectively prevent the development of antifungal resistance. Therefore, finding specific fungicidal leading compounds is an essential task for the development of ideal antifungal drugs (Perlin et al., 2017).

In our previous work, we have synthesized many compounds with different scaffolds and screened antifungal activities by liquid micro-dilution method. (4-phenyl-1, 3-thiazol-2-yl) hydrazine (numbered as 31C), which is similar to the structure of EM-01D2 referred by *Logu etal*, exhibits high *in vitro* antifungal activities against pathogenic fungi such as *Candida, Aspergillus, Cryptococcus*, and *Dermatophytes* (de Logu et al., 2005). Meanwhile, 31C could inhibit the formation of hyphae and biofilms, which is essential for maintaining the virulence of *C. albicans*. In addition, at a concentration of 0.5 μg/ml, 31C showed fungicidal activity against *C. albicans*, which is dependent on the induction of reactive oxygen species (ROS) accumulation. Moreover, 10 mg/kg 31C significantly increased the survival rate of *C. albicans*-infected *Galleria mellonella* (*G. mellonella)*. These results suggested that further structural modification and study of antifungal mechanisms on 31C could provide new directions for the development of antifungal drugs.

## Materials and Methods

### Strains, Mediums, and Agents

*Candida albicans* SC5314, *Cryptococcus neoformans* ATCC32609 and ATCC 34877 are the standard strains used in many fungal studies. Other strains mentioned above are clinical isolates, which were obtained from Changhai Hospital, Shanghai and used in our previous studies (Li et al., 2017, 2019). The fungal strains are usually cultured in YPD liquid medium. Human umbilical vein endothelial cells (HUVECs) were incubated in Dulbecco modified Eagle medium (DMEM) containing 10% fetal bovine serum (FBS). The MIC was evaluated in RPMI 1,640 medium with MOPS. The hyphae formation was tested in RPMI 1,640 and Spider mediums. The peptone, tryptone, yeast extract and agar were purchased from BD. The mineral salts, GSH and TUNEL staining kit were purchased from Meilun (China). The amino acids and DCFH-DA were purchased from Sigma-Aldrich. The Cell Counting Kit-8 was purchased from TargetMol (Shanghai). RPMI 1,640 and FBS were purchased from Gibco.

### Minimal Inhibitory Concentration (MIC) Test

Minimal inhibitory concentration (MIC) mentioned in our study was tested according to the broth microdilution protocol of the Clinical and Laboratory Standards Institute (M27-A3), with a few modifications (Castanheira et al., 2017; Zhong et al., 2017). In brief, *C. albicans* SC5314 or *C. neoformans* was activated in YPD medium for overnight. *Dermatophytes* were cultured on SDA plates and resuspended in RPMI 1,640 for counting. Next, the concentration of fungal suspension in RPMI 1,640 medium was adjusted to 5 × 10^3^ CFU/ml. Then, the fungal suspension and drugs were added to a 96-well plate, and the final concentration of 31C was ranged from 0.017 to 8 μg/ml. The 96-well plates were incubated at 30°C or 35°C for indicated time. *Candida* were cultured for 24 h. *Aspergillus* and *Cryptococcus* were cultured for 72 h. *Dermatophytes* were cultured for 7 days. The growth inhibition was detected by the optical densities at 600 nm (OD_600_). Fluconazole was set as a positive control. The concentration of wells without fungal growth was considered to be MIC.

### Fungicidal Activity Test

The Time-kill studies were performed as previously described (Canton et al., 2013). *C. albicans* SC5314 was cultured in YPD medium overnight and adjusted to 1 × 10^6^ CFU/ml by RPMI 1,640 medium. Different volumes of 31C solution were added to each tube, and the final concentration of 31C was adjusted to 0.0313, 0.0625, 0.125, 0.25, 0.5 μg/ml in each tube. The tubes were cultured at 30°C and different time points. Finally, 100 μl fungal suspensions taken from each tube were placed on the SDA plates, and the fungal clones were counted after 24 h. The time-killing curves were drawn based on the time points and fungal clone numbers.

### The Hyphae Formation Assay

The hyphae formation was determined as previously described (Li et al., 2014). *C. albicans* SC5314 was cultured in YPD medium overnight. The fungal cells were diluted by 1,000 times using Spider or RPMI 1,640 medium. DMSO or 31C solutions were added to the medium at different concentrations. The fungal suspensions with 31C or DMSO were cultured statically at 37°C for 3 h. Finally, the cells were photographed by microscope.

### *In vitro* Biofilm Formation Assay

The biofilm formation assay was performed as described (Zhao et al., 2013; Yang et al., 2019). Briefly, 1 × 10^6^ CFU/ml of *C. albicans* cells in RPMI 1,640 medium were added to a 96-well plate. The plate was cultured for 90 min at 37°C. After adhesion, the RPMI 1,640 medium and non-adherent cells were removed. Then, 100 μl fresh RPMI 1,640 with DMSO or 31C at different concentrations was added. The plate was further inoculated at 37°C for 24 h until the mature biofilms were formed. At last, the mature biofilms were cultured for 3 h with 0.5 mg/ml 2,3-bis-(2- methoxy-4-nitro-5-sulfophenyl)-2H-tetrazolium-5-carboxanilide (XTT) reduction at 37°C. The OD_490_ was detected by the TECAN Infinite M200.

### Cytotoxicity Assay

Cytotoxicity of 31C was assessed by cell counting kit-8 (CCK-8) as described (Lu et al., 2020). HUVECs were cultured in DMEM mediums containing 10% fetal bovine serum (FBS) and used to evaluate the toxicity of 31C. 1 × 10^5^ cells/ml of HUVECs were seeded in 96-well tissue culture plates and incubated for 3 h. After inoculation, different concentrations of 31C dissolved in DMSO were added. Then, the plates were cultured for additional 24 h at 37°C with 5% CO_2_. After inoculation, 10 μl of CCK-8 solution was added to each well, and the HUVECs were incubated for another 2 h. Cell viability was assessed by detecting absorbance at 450 nm. Cells incubated with DMSO treatment were calculated as the standard for 100% viability. Three independent experiments were conducted.

### The ROS Production Assay

Intracellular ROS was detected as described previously (Haque et al., 2019; Jia et al., 2019). *C. albicans* SC5314 cells were stained with 2,7-dichlorfluorescin diacetate (DCFH-DA) to measure the endogenous ROS as described previously. Briefly, saturated cultures of *C. albicans* SC5314 cells were diluted to 1:100 in YPD broth and treated with 31C for 3 h at 30°C. Then, the cells were washed with PBS three times. Next, DCFH-DA was added, whose final concentration was 10 μM in PBS. Fungal cells were inoculated at 30°C for 30 min, and the samples were quantitatively analyzed by flow cytometer.

### Real-Time RT PCR

The expression of fungal genes was determined as described previously (Li et al., 2013b). In brief, *C. albicans* SC5314 cells were treated with 1 μg/ml 31C or DMSO at 30 °C for 3 h. Then, the cells were collected and washed with PBS three times, and the total RNA was isolated by a fungal RNAout kit (60305-50; TIANZ). The concentration of total RNA was measured by NANODROP 2000 (Thermo Fisher), and 500 ng RNA was used for transcription. cDNA was reversely transcripted with a reverse transcription kit (RR037A; TaKaRa Biotechnology). The primers of real-time PCR are shown in the Supplemental Table 1. 1 μl cDNA templates, 0.5 μl forward primers and 0.5 μl reverse primers were added to the reaction system. The expression of each gene was normalized to that of Act-1. The relative expression of each target gene was calibrated against the corresponding expression by untreated *C. albicans* cells, which served as the control.

### TUNEL Staining Assay

DNA breaks were analyzed by the TUNEL method as described (Shirtliff et al., 2009; Yun et al., 2016). Briefly, the *C. albicans* cells were treated with 1 μg/ml 31C at 30°C for 12 h and washed with PBS. Then the cells were fixed in 3.6% paraformaldehyde. Fixed cells were washed twice in PBS. The fixed cells were treated with 500 μl PBS containing 5 μl zymolase (2 U/μl) and inculated at 37°C for 30 min. The cells were washed with PBS and resuspended with buffer containing 0.1% Triton X-100 and 0.1% sodium citrate. After treated for 5 min, the cells were stained as described in Meilun One Step TUNEL Apoptosis Assay Kit (FITC).

### *Galleria mellonella* Infection Assay

The *Galleria mellonella* (*G. mellonella*) fungal infection model was performed as described previously (Li et al., 2013a). Fifteen larvae with an average weight of 180–200 mg were cultured at 37°C overnight before the experiment in each group. Fungal suspensions of *C. albicans* SC5314 were prepared in PBS at a final concentration of 1 × 10^8^. Furthermore, 5 μl fungal suspension or PBS was injected with a Hamilton syringe. 31C was solved with 1% DMSO +35% PEG 400 + 64% ddH_2_O. Larvae infected with *C. albicans* were injected with 5 μl 31C (5 and 10 mg/kg) or drug solutions once. All larvae were incubated at 37°C for 10 days. The death of *G. mellonella* was assessed daily by the lack of movement. For the fungal burden analysis, 5 larvae were homogenized 24 h after administration. Serial 100–10000-fold dilutions were plated on SDA agar and incubated at 30°C for 48 h to enumerate the total fungal burden. Two independent experiments were performed.

## Results

### 31C Exhibited Fungistatic and Fungicidal Activities With Low Toxicity to Mammalian Cells

**Figure 1:**
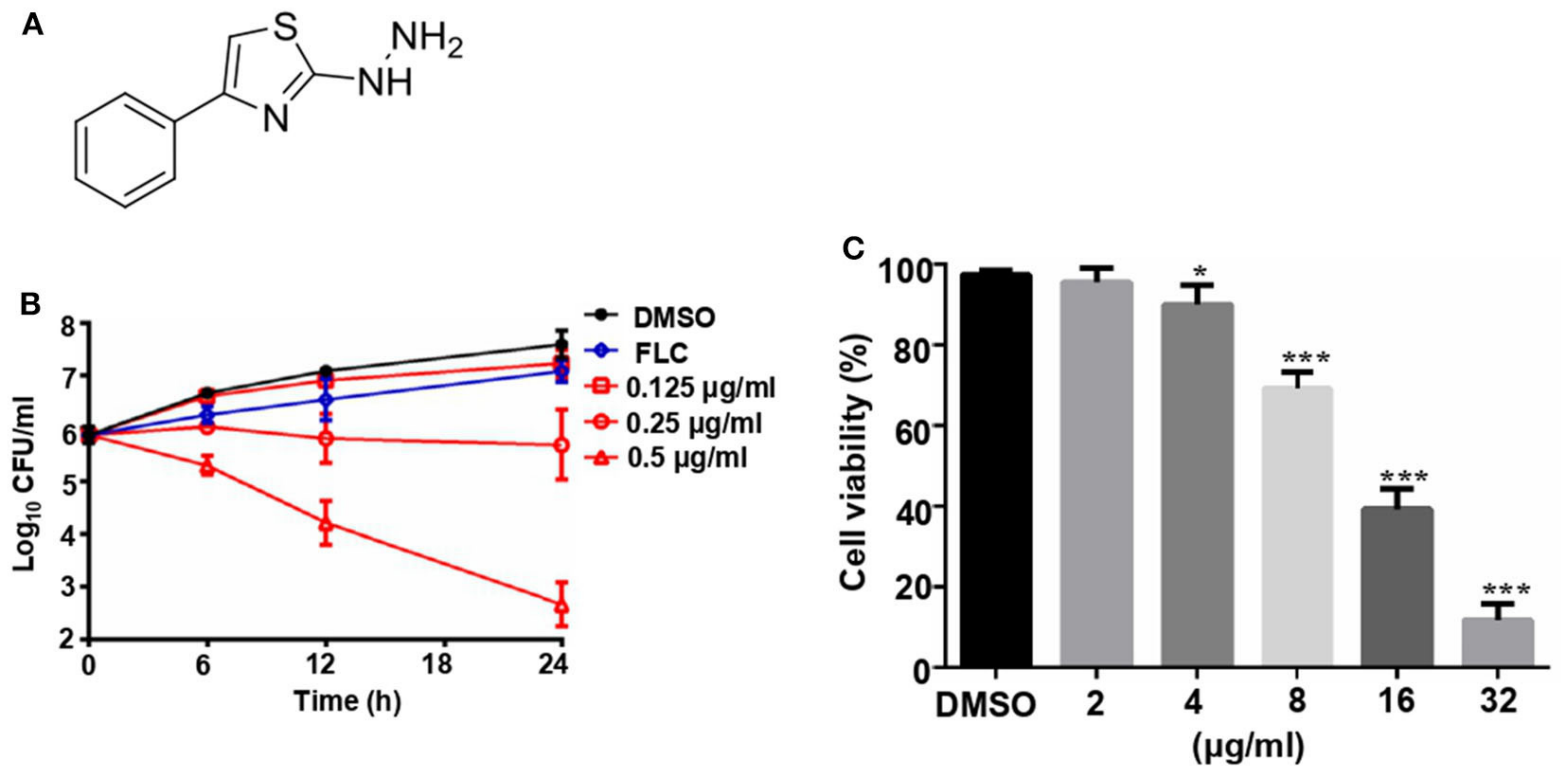
The fungicidal activity and toxity of 31C. **(A)** Chemical structure of (4-phenyl-1, 3-thiazol-2-yl) hydrazine (numbered as 31C). **(B)** Time-killing curves. Log_10_ of CFU/ml remaining after drug exposure in the presence of DMSO, 8 μg/ml fluconazole (FLC), 0.125 μg/ml, 0.25 μg/ml or 0.25 μg/ml 31C in RPMI 1,640 medium were counted. The reported error is SD with *n* = 3. **(C)** Toxicity of 31C determined by cell counting kit-8 in HUVECs. 5 × 10^4^ HUVECs were treated with DMSO, 2, 4, 8, 16, or 32 μg/ml 31C for 24 h in 96-well plates. Similar results were obtained from three independent experiments. *P < 0.05; ***P < 0.001 (Student's *t*-test).

The structure of compound 31C is shown in Figure 1A. During the process of preliminary screening, we found that the minimum inhibitory concentration (MIC) of 31C against *C. albicans* SC5314 was 0.0625 μg/ml. Next, we expanded the antifungal spectrum, and the results showed that MIC of 31C against other common pathogenic fungi was ranged from 0.0625-4 μg/ml, which contains *Candida, Aspergillus, Cryptococcus* and *Dermatophytes* (Table 1). To further ensure that antifungal effect of 31C is not strain-specific, we tested ten *C. albicans* clinical isolates including fluconazole resistant and sensitive strains. As expected, 31C exhibited similar antifungal effect on all the strains, and the MIC is listed in Table 2. These results demonstrated that the broad-spectrum antifungal compound 31C has the potential to be used for the treatment of azole-resistant *C. albicans* infection. In order to examine whether 31C has fungicidal activities, we treated *C. albicans* SC5314 with different concentrations of 31C, and the CFU remaining was counted at different time points to draw the time-killing curves. The result showed that 31C at a concentration of 0.5 μg/ml could effectively kill *C. albicans* SC5314 for 6 h. Nearly 99% *C. albicans* was killed after being treated with 31C of 0.5 μg/ml for 24 h (Figure 1B). To investigate the selectivity of 31C, we assessed its toxicity to human umbilical vein endothelial cells (HUVECs) using cell counting kit-8 (CCK-8). As shown in Figure 1C, no significant influence on the viability of HUVECs was observed when the concentration of 31C was below 4 μg/ml. The 50% inhibitory concentration (IC_50_) of 31C tested by HUVECs was between 8 and 16 μg/ml. Collectively, these results demonstrated that 31C had strong fungistatic and fungicidal activities with low toxicity to mammalian cells, which indicated the high antifungal selectivity of 31C.

**Table 1:** *In vitro* antifungal activities of 31C against fungi (MIC, μg/mL, 24 h).

**Species**	**Isolate**	**MIC (μg/mL)**	
		**31C**	**Voriconazole**
*C. albicans*	SC5314	0.0625	0.0625
*C. krusei*	2,519	0.25	0.5
	4,776	0.125	0.5
*C. glabrata*	537	1	0.25
	8,535	2	0.125
*C. tropicalis*	8,915	0.125	0.0625
	2,718	0.125	2
*C. parapsilosis*	22,019	0.125	0.016
	90,018	0.125	0.016
*A. fumigatus*	023	4	0.125
	7,544	4	0.25
*C. neoformans*	ATCC 34877	0.0625	0.0625
	ATCC 32609	0.0625	0.031
*T. rubrum*	tiA	0.25	2
	tiE	1	1
*M. gypseum*	mza	2	1
	mzc	0.25	0.0625

**Table 2:** *In vitro* antifungal activities of 31C against fluconazole-resistant or fluconazole-sensitive *C. albicans* isolates (MIC, μg/mL, 48 h).

***C. albicans* fluconazole-resistant Isolate**	**MIC (μg/mL)**	
	**31C**	**Fluconazole**
862	0.125	>64
786	0.125	>64
898	0.125	>64
100	0.0625	>64
305	0.0625	>64
379	0.125	>64
385	0.0625	>64
454	0.25	>64
504	0.0625	>64
Y0109	0.0625	0.0313

### 31C Inhibited the Formation of *C. albicans* Hyphae and Biofilms

**Figure 2:**
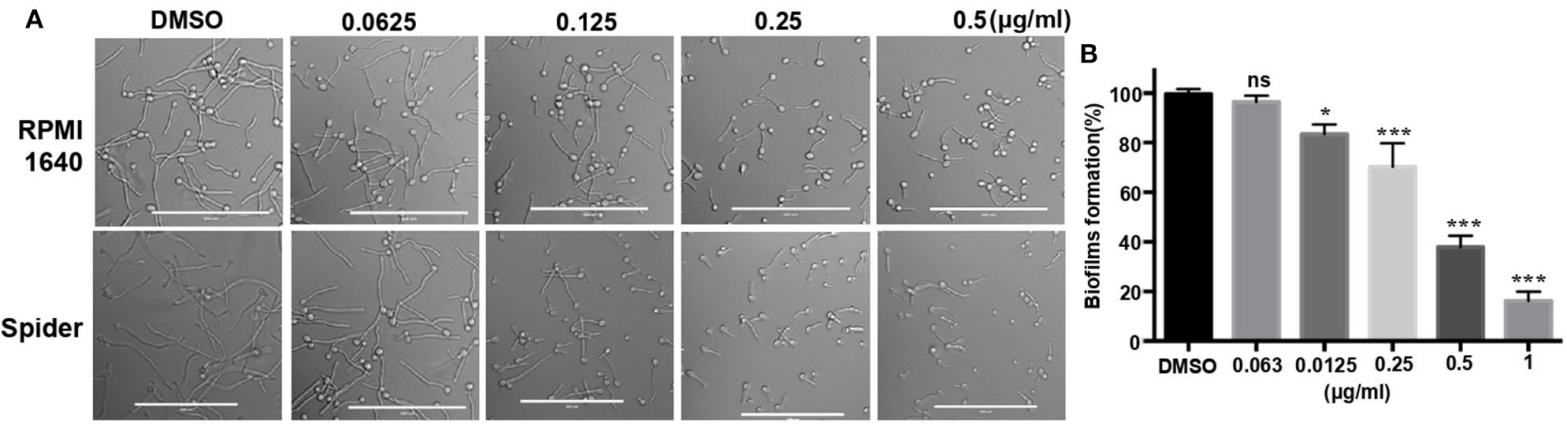
Hyphae and biofilms formation inhibited by 31C. **(A)** Hyphae formation inhibition. Exponentially growing *C. albicans* SC5314 cells were cultured in hyphae-inducing RPMI 1,640 or Spider liquid medium at 37°C for 3 h. Bars, 100 μm. **(B)** Biofilm formation detected by XTT reduction assay. *C. albicans* SC5314 were cultured with 0.063-1 μg/ml 31C in 96-well polystyrene plate at 37°C for 24 h. The results represent means standard deviations for three independent experiments. *P < 0.05; ***P < 0.001 (Student's *t*-test).

In addition to studying the growth inhibition of 31C, we also investigated its effects on the hyphae and biofilms formation, which is associated with the virulence of *C. albicans* (Soll and Daniels, 2016). The result showed that hyphae formation was inhibited by 0.125 μg/ml of 31C in RPMI 1,640 and Spider medium partially. Additionally, the inhibitory effect was dose-dependent as shown in Figure 2A. Similarly, 31C also inhibited the formation of biofilms determined by a 2, 3-bis-(2-methoxy-4-nitro-5-sulfophenyl)-2H-tetrazolium-5-carboxanilide (XTT) reduction assay. As shown in Figure 2B, 31C of 0.25 μg/ml inhibited biofilms formation by about 30% (*P* < 0.001), and the suppressive effect was more significant as the 31C concentration increased. Overall, these results further illustrated the powerful antifungal effects of 31C.

### Antifungal Effects of 31C Against *C. albicans in vivo*

**Figure 3:**
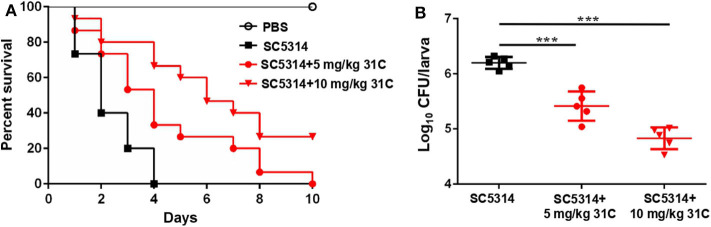
The survival of *G. mellonella* larvae infected with *C. albicans*. **(A)** Survival curve of *G. mellonella* (*n* = 15). **(B)** The fungal burden of each larva (*n* = 5) infected for 24 h. Each larva was infected with 5 × 10^5^ CFU SC5314 and treated with 31C (5 and 10 mg/kg) once. Similar results were obtained from two independent experiments. ***p < 0.001, ns, not significant, by log rank (Mantel-Cox) test.

*G. mellonella* was a described mini-host for Candida species by Cotter et al. (2000). In addition, the *G. mellonella* larvae can be incubated at 37°C, which is an important factor that influences the virulence of *C. albicans in vivo*. In recent years, the fungal infection models of *G. mellonella* have been widely used in the evaluation of fungal virulence and antifungal drug efficiency, including *Candida, Cryptococcus, Trichosporon, Aspergillus* and *Mucorales* (Ames et al., 2017; Gong et al., 2019; Trevino-Rangel et al., 2019; Jemel et al., 2020). We tested the protective effect of 31C using the fungal infection model of *G. mellonella*. After the incubation with 5 mg/kg or 10 mg/kg 31C, the survival time of *G. mellonella* was prolonged (Figure 3A). The survival rate of *G. mellonella* was significantly improved to about 30% with the treatment of 10 mg/kg 31C, which was higher than the solvent control group. Consistently, the fungal burden of *G. mellonella* treated with 31C decreased significantly, which indicated the strong antifungal activities of 31C *in vivo* (Figure 3B).

### 31C Treatment Induced Oxidative-Damage in *C. albicans*

**Figure 4:**
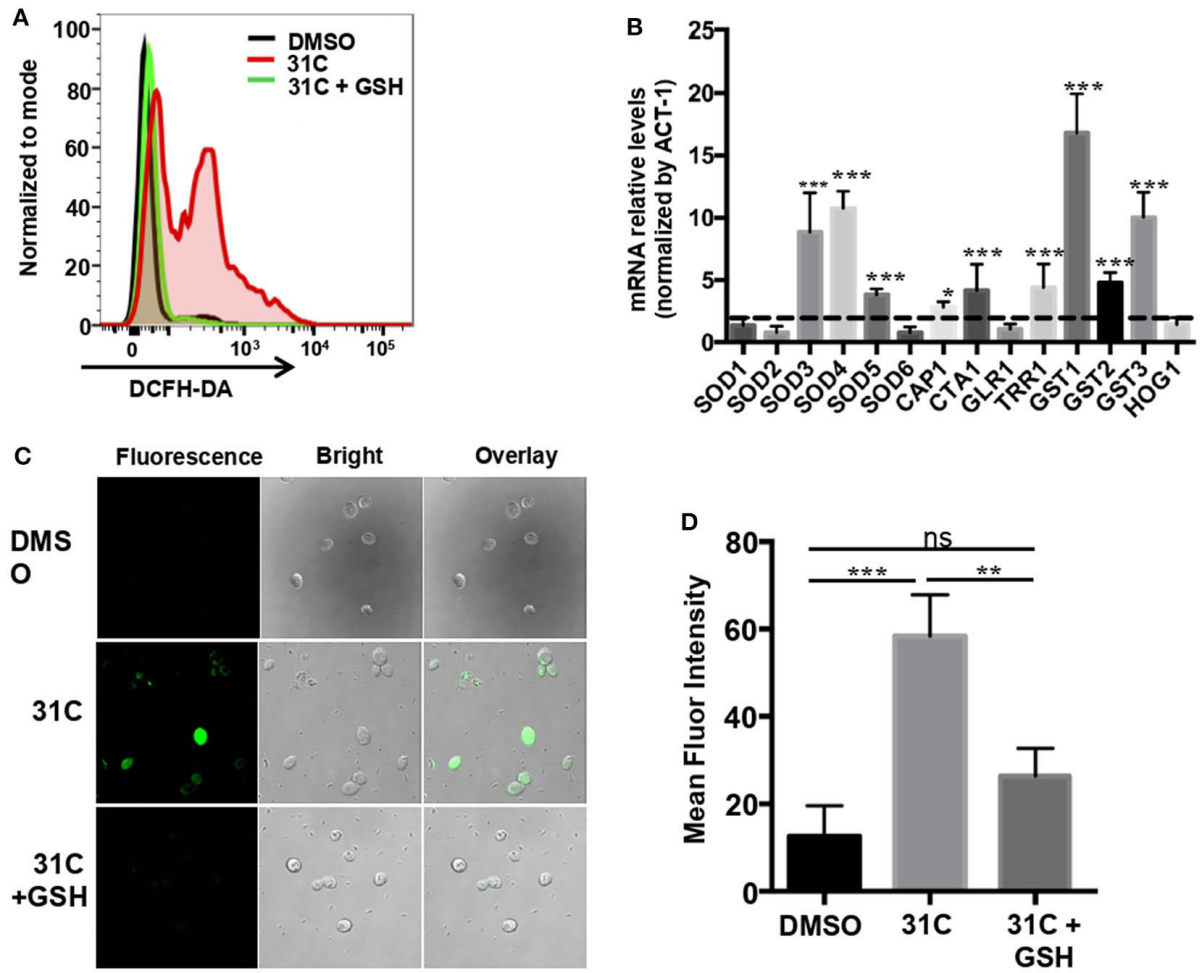
31C treatment elevated the intracellular oxidative stress in *C. albicans*. **(A)** The ROS levels tested by flow cytometry. *C. albicans* SC5314 cells were treated with DMSO, 0.5 μg/ml 31C or 0.5 μg/ml 31C + 10 mM GSH for 6 h. The generation of ROS was measured by 20 μM DCFH-DA. The representative fluorescent intensity image was showed. **(B)** The expression of anti-oxidative genes detected by RT-PCR. *C. albicans* SC5314 cells were treated with DMSO or 0.5 μg/ml 31C for 3 h. *ACT-1* was used to normalize the expression data. The dashed line represents genes up-regulated two-folds. Data were obtained from three independent experiments. **(C)** Cells were stained with TUNEL apoptosis assay kit. The fluorescence of cells was quantified using confocal microscopy **(C)** or flow cytometry **(D)**. Data are shown as means ± standard deviations from triplicate experiments. *P < 0.05; **P < 0.01; ***P < 0.001 (One-way ANOVA test).

In order to further explore the fungicidal mechanism of 31C, we searched the literature and found that the DNA damage caused by the oxidative stress plays a critical role in antifungal-induced cellular death reported by Belenky et al. (2013). Therefore, we speculated that ROS-induced oxidative stress may be a reason for the fungicidal activity of 31C. As shown in Figure 1B, 31C could significantly kill *C. albicans* SC5314 in 6 h. In order to investigate whether the fungicidal activity of 31C depends on oxidative damage, we used the fluorescent dye 2, 7-Dichlorodi-hydrofluorescein diacetate (DCFH-DA) to detect the intracellular ROS levels in *C. albicans*. Flow cytometry analysis showed that 0.5 μg/ml 31C could significantly elevate the intracellular ROS levels (Figure 4A). Adding high levels of ROS scavenger glutathione (GSH) could reduce the intracellular ROS to the naive levels. Meanwhile, we used RT-PCR to detect the expression of genes related with fungal oxidative stress response. The result showed that *SOD3, SOD4*, and *SOD5*, encoding superoxide dismutases, were up-regulated 3–10-folds after being treated with 31C of 0.5 μg/ml for 3 h. Beyond that, *CAP1*, encoding a transcription factor participating in oxidative stress tolerance in *C. albicans*, was also up-regulated. Furthermore, the expression of genes *CTA1, TRR1* which were regulated by *CAP1* and genes *GST1, GST2, GST3* which encoded glutathione S-transferases increased significantly (Figure 4B). These results indicated that 31C treatment induced the occurrence of oxidative stress, which was accompanied with the activated anti-oxidative stress response in *C. albicans*. In previous reports, high levels of ROS could damage multiple cellular targets, including membranes, proteins, DNA, and promote the apoptosis of cells (Ikner and Shiozaki, 2005). To further investigate the cellular damage caused by oxidative stress, we utilized the terminal deoxynucleotidyltransferase-mediated dUTP-biotin nick end labeling (TUNEL) assay to quantify the relative abundance of double-strand DNA breaks in *C. albicans* after 31C treatment. Elevated relative fluorescence was detected in the cells treated with 31C by confocal microscopy (Figure 4C) and flow cytometry (Figure 4D). When the ROS scavenger GSH added, the numbers of apoptotic cells decreased significantly (Figures 4C,D). These results suggested that 31C increased the apoptosis of *C. albicans*, depending on the elevated intracellular oxidative stress.

### ROS Scavengers Significantly Reduced the Antifungal Activity of 31C

**Figure 5:**
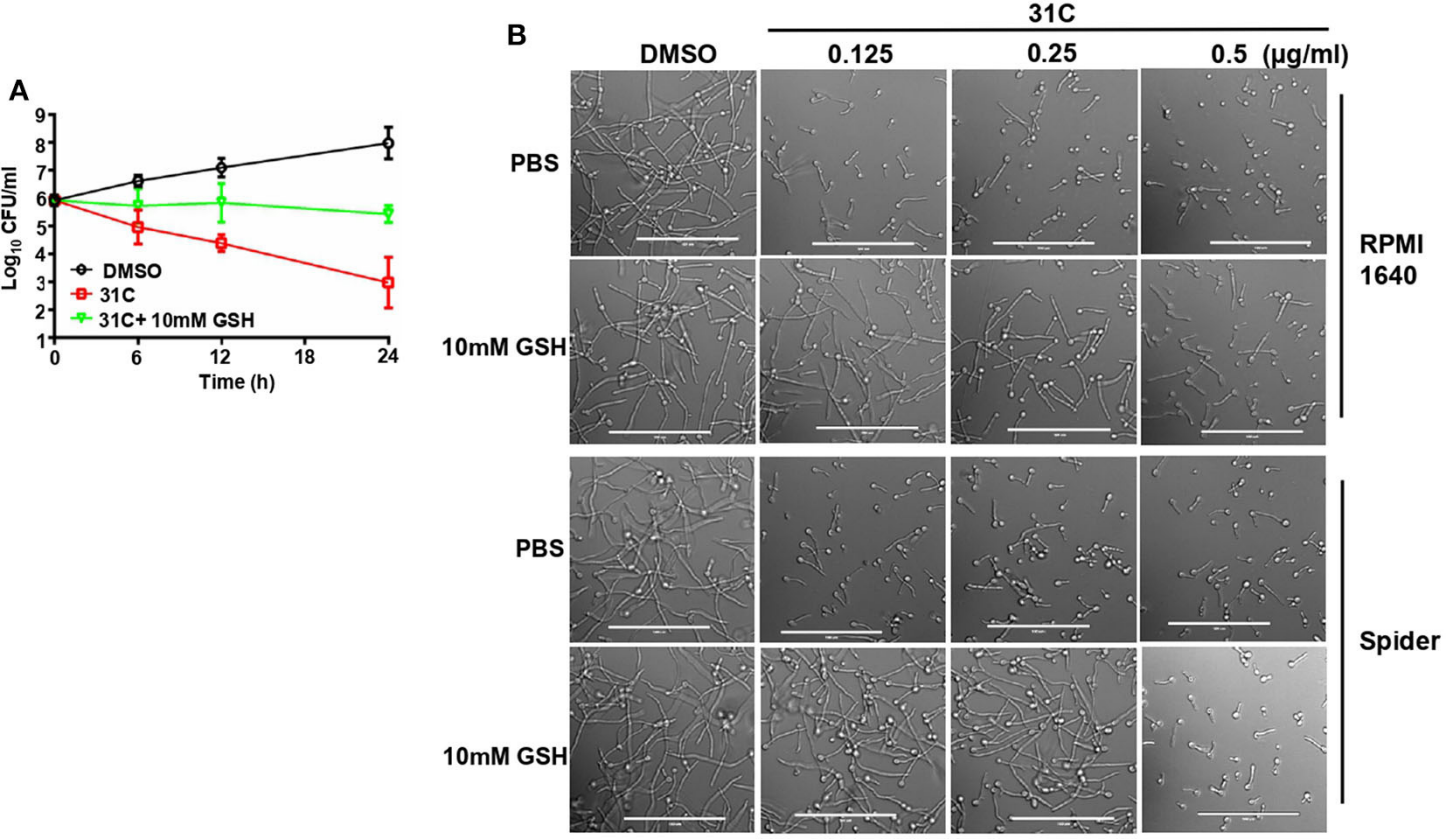
ROS scavenger GSH decreased the antifungal activity of 31C. **(A)** Time-killing curves. Log_10_ of CFU/ml remaining after drug exposure in the presence of DMSO, 0.5 μg/ml 31C or 0.5 μg/ml 31C + 10 mM GSH in RPMI 1640 medium were counted. The reported error is SD with *n* = 3. **(B)** Hyphae formation inhibited by 31C was partially restored by ROS scavenger GSH. Exponentially growing *C. albicans* SC5314 cells were cultured in hyphae-inducing RPMI 1640 or Spider liquid medium at 37 °C for 3 h. 0.125-0.5 μg/ml 31C and 10 mM GSH was added or not. Bars, 100 μm.

To elucidate the role of 31C-induced oxidative stress, we further investigated the effect of ROS scavengers on the antifungal activity of 31C. First, we added some other ROS scavengers, containing N-acetylcysteine (NAC), glutathione and oligomeric proantho cyanidins (OPC), to the RPMI1640 medium to detect the sensitivity of *C. albicans* to 31C. As shown in Table 3, the MIC increased 8–32-folds after addition of ROS scavenger, which indicated that the antifungal activity of 31C was significantly reduced. As control, we compared the effects of oxidized GSSH on the MIC of 31C. The result showed that the addition of 10 mM GSSH had no influence on the tolerance of *C. albicans* to 31C. These results indicated that reducing substances made *C. albicans* resistant to 31C. Meanwhile, we also focused on the role of GSH in the fungicidal activity of 31C, as oxidative-damage is important in the fungal killing mechanism. The time-killing curve showed that exposing *C. albicans* to 10 mM GSH considerably diminished the killing ability of 31C (Figure 5A). In addition, the inhibition of hyphae formation induced by 31C was also partially dependent on the production of ROS. When the concentration of 31C in the spider or RPMI 1,640 medium was lower than 0.25 μg/ml, the addition of 10 mM GSH could restore the hyphae formation of *C. albicans* (Figure 5B). In summary, 31C-induced oxidative damage is an important factor in killing *C. albicans* and inhibiting hyphae formation. Adding ROS scavengers could abrogate the antifungal effects of 31C to some extent. These results confirmed that the antifungal activities of 31C were partially dependent on the intracellular oxidative damage.

**Table 3:** *In vitro* antifungal activities of 31C against *C. albicans* SC5314 in mediums containing different agents (MIC, μg/mL, 48 h).

**Mediums**	**MIC (μg/mL)**
YPD	0.125
YPD + 10 mM GSH	16
YPD + GSSH	0.125
YPD + 5 mM NAC	4
YPD + 2 mM OPC	8

## Discussion

In recent decades, the incidence of invasive fungal infections, mainly including *C. albicans, C. neoformans* and *A. fumigatus* infection, has increased dramatically (Suleyman and Alangaden, 2016). Meanwhile, azoles have been widely used for more than 30 years, and the cross-resistance of fungi has been widely reported (Berman and Krysan, 2020). Therefore, it is imminent to explore highly effective and low-toxic antifungal compounds with new mechanisms. In this study, we identified a new antifungal compound (4-phenyl-1, 3-thiazol-2-yl) hydrazine, numbered as 31C, which is similar to the structure of EM-01D2. However, the research on EM01D2 is only limited to its vitro antifungal activity. The systematic evaluation of antifungal activities and related mechanism studies of such compounds have not been reported. Our research demonstrated the clear and broad-spectrum antifungal activity of 31C. The MIC of 31C against *C. albicans* was as low as 0.0625 μg/ml. When the concentration increased from 0.0625 to 0.5 μg/ml, 31C showed fungicidal activity and inhibited the formation of hyphae and biofilms. *In vitro* cytotoxicity tests by HUVEC cells indicated that 31C was less toxic, as the cytotoxic IC_50_ was 8–16 μg/ml. These results suggested that 31C may specifically target fungal proteins, which are less homologous to mammalian cells. In addition, we also investigated the activity of 31C *in vivo*, and a strong protective effect was observed in the *G. mellonella* infection model.

In our study, the antifungal mechanism of 31C was preliminarily explored, which is partially dependent on the oxidative damage of ROS. 31C-induced ROS production promotes the DNA fragmentation, and this killing effect can be significantly abrogated by ROS scavengers. Recent studies have found that high levels of endogenous ROS in pathogenic fungi could cause apoptosis and irreversible cell damage, such as destroyed proteins, lipids, and nucleic acids (Ribeiro et al., 2006; Dantas Ada et al., 2015). Additionally, our previous studies also indicated that increased intracellular ROS resulted in the filamentation defects, which is consistent with the conclusion that 31C inhibits hyphae formation by inducing oxidative stress in *C. albicans* (Bi et al., 2018). All these studies provide new strategies for studying antifungal drugs. On the one hand, the antifungal effects of many compounds were confirmed to be related to the elevated endogenous ROS. Compounds with different scaffolds, such as shikonin, heterocycle thiazole compounds, clerodane type diterpene, and SM21, exhibited antifungal activity through increasing the production of ROS in fungi (Miao et al., 2012; Bhattacharya et al., 2015; de Sa et al., 2017; Truong et al., 2018). On the other hand, the induction of elevated endogenous ROS is the mechanism of many antifungal synergists. Honokiol or tetrandrine could significantly reduce the fluconazole resistance of *C. albicans* by increasing the intracellular ROS (Guo et al., 2014; Sun et al., 2017). Therefore, elucidating the mechanism of drug-induced ROS production or accumulation is of great significance for the development of antifungal drugs (Li et al., 2015).

Our studies showed that 31C treatment elevated the ROS levels in *C. albicans*. However, it is unclear how 31C treatment affects the generation or elimination of ROS. As reported, mitochondria were considered to be the major source of ROS (Helmerhorst et al., 2002). The ROS is usually generated from electron leakage originating in the mitochondrial transport chain during respiration. The ROS production in the mitochondrial chain always includes the NADH dehydrogenases Nde1p and Nde2p and complex III. In normal conditions, intracellular superoxide dismutase, catalase and various peroxidases could reduce the cellular damage caused by ROS (Farrugia and Balzan, 2012; Nickel et al., 2014). When intracellular ROS is elevated, *C. albicans* will initiate effective oxidative stress response regulated by transcription factor Cap1p or Hog1p (Enjalbert et al., 2006; Wang et al., 2006). Cap1p rapidly aggregates in the nucleus and regulates a series of gene transcription, such as *CTA1, GLR1, TRR1* (Kusch et al., 2007; Dantas et al., 2010). In our study, gene expression analysis indicated that 31C treatment unregulated the transcription of antioxidant proteins, such as Cap1p, superoxide dismutases and glutathione S-transferases. However, the mechanism of intracellular ROS accumulation induced by 31C is not clear at the present stage.

Finally, our research has found a new broad-spectrum, highly effective and low-toxic antifungal compound. The structural modification based on 31C could provide new ideas for the development of antifungal drugs.

## Data Availability Statement

All datasets presented in this study are included in the article/Supplementary Material.

## Author Contributions

Q-ZL, T-J-HN, Y-YJ, and D-ZZ conceived and designed the study. L-PL and TL performed data analysis. Q-ZL wrote the manuscript. Q-ZL, T-J-HN, L-PL, and TL conducted all the experiments and performed the statistical analysis of the data. All authors contributed to the article and approved the submitted version.

## Conflict of Interest

The authors declare that the research was conducted in the absence of any commercial or financial relationships that could be construed as a potential conflict of interest.
