# Supplementary material for: A New Antifungal Agent (4-phenyl-1, 3-thiazol-2-yl) Hydrazine Induces Oxidative Damage in Candida albicans
Source: Front Cell Infect Microbiol. 2020 Oct 7;10:578956. doi: 10.3389/fcimb.2020.578956 (PMC7575736; doi:10.3389/fcimb.2020.578956)
Supplement: Supplementary file 1 [file Data_Sheet_1.PDF]

Table S1. Primers used in RT-PCR.

Genes	Primers	Genes	Primers
SOD1-F	AGGGTCTTGGTTCCAACCTT	CAP1-F	GAACCCCCAAAAGCAGAACT
SOD1-R	CCTCTGACAACAGCGACTGA	CAP1-R	CTTGTTCGACAAATGCCTCA
SOD2-F	TTAAAGGCTTCTTCCGCAAC	GLR1-F	GATCTGGTGGTGTTGCCTCT
SOD2-R	GCTTCAACGGCTTGTTCAAT	GLR1-R	TTAGCCCAATCGAAATCTCC
SOD3-F	ATTGATTGGGCTCTTGATGC	GST1-F	GCCCAAAAAGAAACGGGTAT
SOD3-R	CGACTTGAGATCCCTCTTGC	GST1-R	AGTTGGAACAAGGCCATGAG
SOD4-F	AGGCAAGGCACCATTAGTTG	GST2-F	CACATACGGAACTCCAAACG
SOD4-R	CAGCTGGTGTTTTGGCAGTA	GST2-R	GCACCGGTTTGACTAATGGT
SOD5-F	CTCCAAAGGCAGTCCATCAT	GST3-F	TTTCATGGCCCACTAAAGAT
SOD5-R	GTTGCAGCTCTAACGGTTCC	GST3-R	CCAATACTCCAAGGGGTCAG
SOD6-F	AGGTCAAAGGAAACGTGGTG	HOG1-F	GAATGGGAGCATTTGGTTTG
SOD6-R	CATCGCAAACTGGACTAGCA	HOG1-R	CCTATGCAAATCAGTACCTTGC
CTA1-F	ACCCAGATTACGCCCAAGAA	TRR1-F	TCATTGCTACTGGTGCCTCT
CTA1-R	GGGGAGAAAGCAGCTTGTTC	TRR1-R	CAAGCTGAATCACCACCACC
